# Poly[(μ_3_-*rac*-5-eth­oxy­carbonyl-6-hy­droxy-6-methyl-4-phenyl-4,5,6,7-tetra­hydro­benzo[*c*]isoxazol-3-olato)potassium]

**DOI:** 10.1107/S160053681101868X

**Published:** 2011-05-25

**Authors:** Abel M. Maharramov, Arif I. Ismiyev, Bahruz A. Rashidov

**Affiliations:** a Baku State University, Z. Khalilov St. 23, Baku AZ-1148, Azerbaijan

## Abstract

The title compound, [K(C_17_H_18_NO_5_)]_*n*_, reveals the relative configuration (4R*,5S*,6R*) whereas its crystals are racemic. The cyclo­hexane ring adopts a half-chair conformation and the isoxazole ring has an envelope conformation. The ethyl fragment of the eth­oxy­carbonyl group at position 5 is disordered in a 0.547 (7):0.453 (7) ratio. The K^+^ ion is surrounded by five O atoms from three ligands at distances ranging from 2.606 (2) to 3.028 (2) Å, generating a three-dimensional network. The crystal packing displays inter­molecular O—H⋯N and O—H⋯O hydrogen bonds in which the hy­droxy group acts as a double proton donor.

## Related literature

For background to the microbiological activity of 2-azetidin­one derivatives, see: Wadher *et al.* (2009 ▶). 
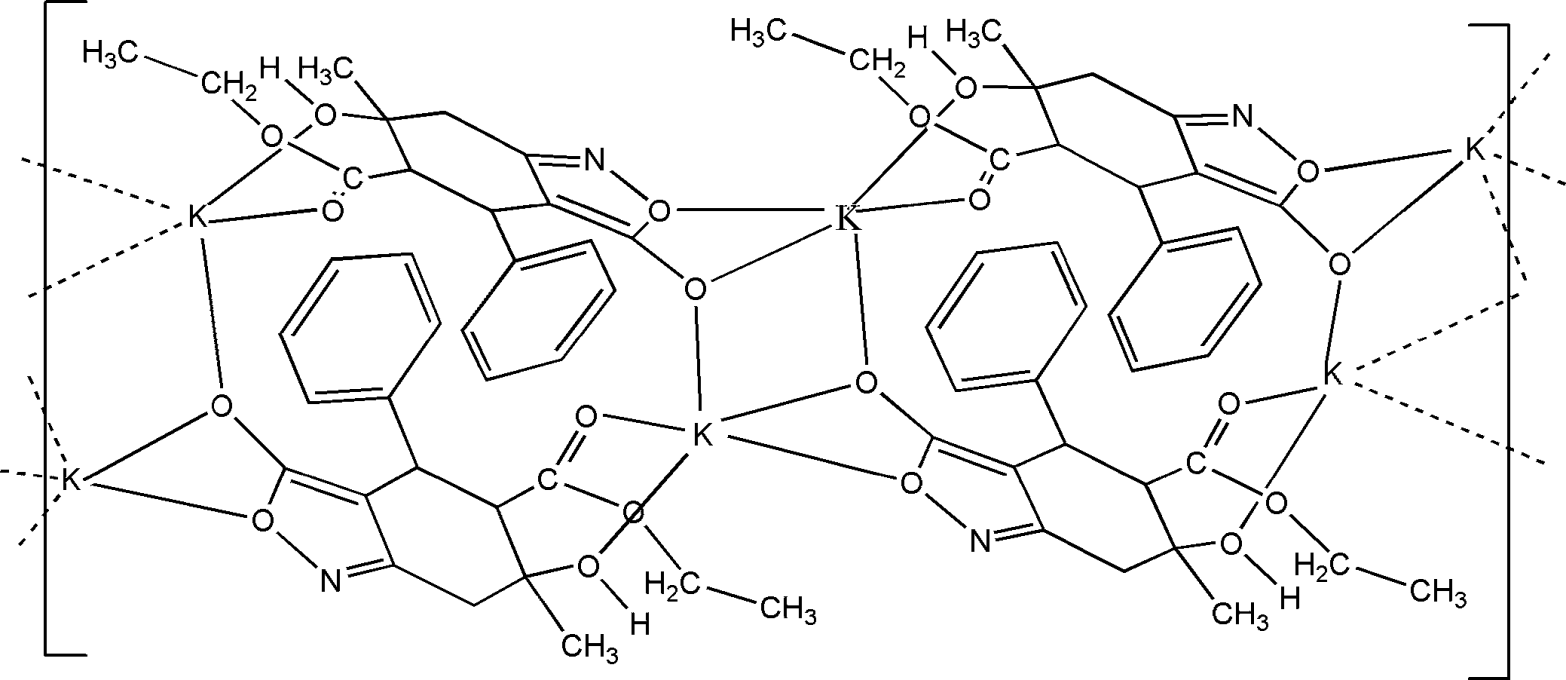


## Experimental

### 

#### Crystal data

[K(C_17_H_18_NO_5_)]
*M* *_r_* = 355.42
Monoclinic, 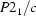

*a* = 12.4811 (18) Å
*b* = 15.411 (2) Å
*c* = 8.6647 (12) Å
β = 94.388 (5)°
*V* = 1661.7 (4) Å^3^
*Z* = 4
Mo *K*α radiation
μ = 0.35 mm^−1^
*T* = 100 K
0.30 × 0.30 × 0.20 mm

#### Data collection

Bruker APEXII CCD diffractometer
Absorption correction: multi-scan (*SADABS*; Sheldrick, 1998 ▶) *T*_min_ = 0.903, *T*_max_ = 0.934
16716 measured reflections
3594 independent reflections
2977 reflections with *I* > 2σ(*I*)
*R*_int_ = 0.042

#### Refinement

*R*[*F* ^2^ > 2σ(*F* ^2^)] = 0.054
*wR*(*F* ^2^) = 0.149
*S* = 0.99
3594 reflections
221 parameters
6 restraints
H-atom parameters constrained
Δρ_max_ = 0.68 e Å^−3^
Δρ_min_ = −0.64 e Å^−3^

### 

Data collection: *APEX2* (Bruker, 2005 ▶); cell refinement: *SAINT-Plus* (Bruker, 2001 ▶); data reduction: *SAINT-Plus*; program(s) used to solve structure: *SHELXTL* (Sheldrick, 2008 ▶); program(s) used to refine structure: *SHELXTL*; molecular graphics: *SHELXTL*; software used to prepare material for publication: *SHELXTL*.

## Supplementary Material

Crystal structure: contains datablocks global, I. DOI: 10.1107/S160053681101868X/kp2318sup1.cif

Structure factors: contains datablocks I. DOI: 10.1107/S160053681101868X/kp2318Isup2.hkl

Additional supplementary materials:  crystallographic information; 3D view; checkCIF report

## Figures and Tables

**Table 1:** Hydrogen-bond geometry (Å, °)

*D*—H⋯*A*	*D*—H	H⋯*A*	*D*⋯*A*	*D*—H⋯*A*
O6—H6*O*⋯N1^i^	0.91	1.88	2.784 (3)	177
O6—H6*O*⋯O2^i^	0.91	2.55	3.336 (3)	145

Symmetry code: (i) 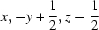.

**Fig. 1.**
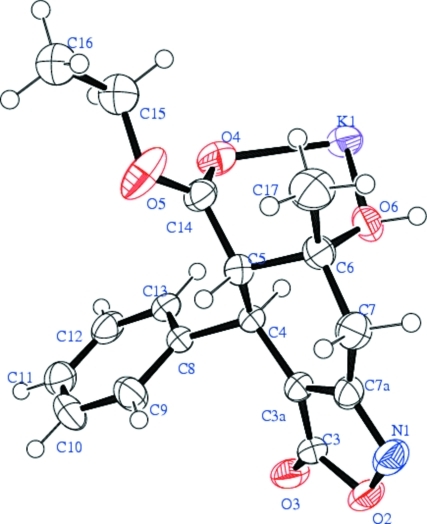
The molecular structure of the title compound with the atomic numbering scheme. Displacement ellipsoids were drawn at the 30% probability level.

**Fig. 2.**
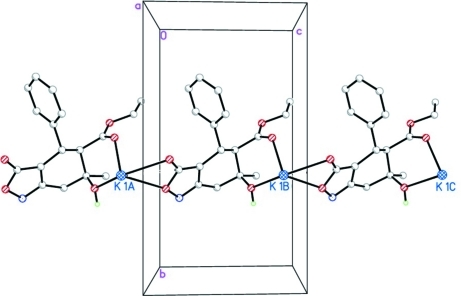
The polymeric structure of the title compound.

**Fig. 3.**
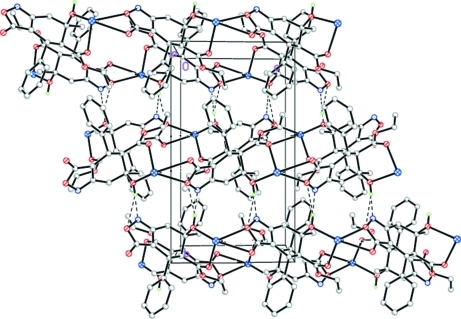
The hydrogen-bonded (dashed lines) packing in the title compound. H atoms not involved in hydrogen bonding have been omitted for clarity.

## supplementary crystallographic information

### Comment

Schiff-base compounds have been used as fine chemicals and medical substrates. They are associated with antibacterial, antifungal and antitubercular activities and have diverse biological activities. Literature revealed that 2-azetidinone derivatives occupy an important place in medicinal chemistry as they show a variety of microbiological activity (Wadher *et al.*2009).

The molecules (I) are diastereomers and possess three asymmetric centers at C4, C5 and C6 carbon atoms. The crystal of (I) is racemate and consists of enantiomeric pairs with the relative configuration of the centres of *rac*-4*R*,5S*,6R*.* The cyclohexane ring adopts a half-chair conformation (Fig. 1). Izoxazole ring has an envelope conformation. The fragment of a ring C7a—N1—O2—C3 is almost planar - torsion angle is 1.0 (2) °. The phenyl ring is in a pseudo-equatorial position. Torsion angle between the ethoxycarbonyl group and the phenyl substituent is C8—C4—C5—C14 is -61.3 (2)° which indicates a pseudo-axial location of hydrogen atoms at C4 and C5. K+ creates coordination with the contacts: from the same ligand K1···O4–2.688 (2) and K1···O6–2.804 (2)Å; from one ligand K1···O3–2.606 (2)%A [-x,1-y, 1-z] and with an additional ligand K1···O3 2.701 (2) and K1···O2 3.028 (2)Å [x,y,-1+z]. O3 atoms form double bridges  between the two K+ (Fig. 2) The closest contac K1—C12 is 3.372 (2) Å. The crystal structure involves O—H···N and O—H···O hydrogen bonds (Table 1 and Fig. 3).

### Experimental

(rac)-Diethyl-4-hydroxy-4-methyl-6-oxo-2-phenyl-1,3-dicarboxylate (20 mmol), hydroxylamine hydrochloride (20 mmol) were dissolved in 20 mL ethanol. The mixture was stirred at 345–350 K for 10 min. Then added 40 (mmol) potassium bicarbonate and continued with mixing up to 10 h. After cooling to a room temperature white crystals were obtained. The crystals were filtered and washed with ethanol. Then they were dissolved in ethanol (50 mL) and recrystallised to yield colourless block-shaped crystals for structure determination.

### Refinement

The hydrogen atoms of the NH and OH-groups (I) molecule were localised in the difference-Fourier map and included in the refinement with fixed positional and isotropic displacement parameters [*U*_iso_(H) = 1.5*U*_eq_(C) for CH_3_-group and *U*_iso_(H) = 1.2*U*_eq_(N) for amino groups]. The other hydrogen atoms were placed in calculated positions with and refined in the riding mode with fixed isotropic displacement parameters [*U*_iso_(H) = 1.2*U*_eq_(C)].

### Crystal data

**Table:**

[K(C_17_H_18_NO_5_)]	*F*(000) = 744
*M**_r_* = 355.42	*D*_x_ = 1.421 Mg m^−^^3^
Monoclinic, *P*2_1_/*c*	Mo *K*α radiation, λ = 0.71073 Å
Hall symbol: -P 2ybc	Cell parameters from 2419 reflections
*a* = 12.4811 (18) Å	θ = 2.7–32.7°
*b* = 15.411 (2) Å	µ = 0.35 mm^−^^1^
*c* = 8.6647 (12) Å	*T* = 100 K
β = 94.388 (5)°	Prism, colorless
*V* = 1661.7 (4) Å^3^	0.30 × 0.30 × 0.20 mm
*Z* = 4	

### Data collection

**Table:**

Bruker APEXII CCD diffractometer	3594 independent reflections
Radiation source: fine-focus sealed tube	2977 reflections with *I* > 2σ(*I*)
graphite	*R*_int_ = 0.042
φ and ω scans	θ_max_ = 27.0°, θ_min_ = 1.6°
Absorption correction: multi-scan (*SADABS*; Sheldrick, 1998)	*h* = −15→15
*T*_min_ = 0.903, *T*_max_ = 0.934	*k* = −19→19
16716 measured reflections	*l* = −11→11

### Refinement

**Table:**

Refinement on *F*^2^	Primary atom site location: structure-invariant direct methods
Least-squares matrix: full	Secondary atom site location: difference Fourier map
*R*[*F*^2^ > 2σ(*F*^2^)] = 0.054	Hydrogen site location: difference Fourier map
*wR*(*F*^2^) = 0.149	H-atom parameters constrained
*S* = 0.99	*w* = 1/[σ^2^(*F*_o_^2^) + (0.080*P*)^2^ + 1.240*P*] where *P* = (*F*_o_^2^ + 2*F*_c_^2^)/3
3594 reflections	(Δ/σ)_max_ = 0.001
221 parameters	Δρ_max_ = 0.68 e Å^−^^3^
6 restraints	Δρ_min_ = −0.64 e Å^−^^3^

### Special details

**Table:**

Geometry. All e.s.d.'s (except the e.s.d. in the dihedral angle between two l.s. planes) are estimated using the full covariance matrix. The cell e.s.d.'s are taken into account individually in the estimation of e.s.d.'s in distances, angles and torsion angles; correlations between e.s.d.'s in cell parameters are only used when they are defined by crystal symmetry. An approximate (isotropic) treatment of cell e.s.d.'s is used for estimating e.s.d.'s involving l.s. planes.
Refinement. Refinement of *F*^2^ against ALL reflections. The weighted *R*-factor *wR* and goodness of fit *S* are based on *F*^2^, conventional *R*-factors *R* are based on *F*, with *F* set to zero for negative *F*^2^. The threshold expression of *F*^2^ > σ(*F*^2^) is used only for calculating *R*-factors(gt) *etc*. and is not relevant to the choice of reflections for refinement. *R*-factors based on *F*^2^ are statistically about twice as large as those based on *F*, and *R*- factors based on ALL data will be even larger.

### Fractional atomic coordinates and isotropic or equivalent isotropic displacement parameters (Å^2^)

**Table:**

	*x*	*y*	*z*	*U*_iso_*/*U*_eq_	Occ. (<1)
K1	0.05763 (5)	0.40629 (3)	0.14073 (6)	0.04742 (19)	
N1	0.28041 (17)	0.31949 (12)	0.8020 (2)	0.0439 (5)	
O2	0.19438 (14)	0.35565 (10)	0.88107 (18)	0.0439 (4)	
C3	0.15026 (18)	0.42478 (13)	0.7957 (2)	0.0343 (4)	
O3	0.07151 (14)	0.46143 (11)	0.84704 (18)	0.0440 (4)	
C3A	0.20830 (16)	0.43430 (12)	0.6674 (2)	0.0308 (4)	
C4	0.19038 (16)	0.49360 (12)	0.5307 (2)	0.0276 (4)	
H4	0.1313	0.4679	0.4608	0.033*	
C5	0.29248 (17)	0.49356 (13)	0.4414 (2)	0.0323 (4)	
H5	0.3515	0.5229	0.5063	0.039*	
O4	0.19551 (16)	0.53784 (12)	0.2040 (2)	0.0537 (5)	
O5	0.35688 (16)	0.59290 (14)	0.2631 (3)	0.0684 (6)	
C6	0.32838 (17)	0.39892 (13)	0.4075 (3)	0.0344 (4)	
O6	0.23646 (12)	0.35629 (9)	0.33610 (18)	0.0389 (4)	
H6O	0.2512	0.2992	0.3219	0.058*	
C7	0.36413 (18)	0.35424 (14)	0.5608 (3)	0.0399 (5)	
H7A	0.3724	0.2912	0.5430	0.048*	
H7B	0.4348	0.3775	0.6007	0.048*	
C7A	0.28494 (17)	0.36821 (13)	0.6776 (2)	0.0343 (4)	
C8	0.15431 (16)	0.58394 (12)	0.5748 (2)	0.0296 (4)	
C9	0.2076 (2)	0.63132 (15)	0.6927 (3)	0.0455 (6)	
H9	0.2712	0.6087	0.7452	0.055*	
C10	0.1689 (3)	0.71179 (16)	0.7348 (3)	0.0551 (7)	
H10	0.2067	0.7440	0.8152	0.066*	
C11	0.0769 (2)	0.74512 (15)	0.6616 (3)	0.0499 (6)	
H11	0.0510	0.8003	0.6907	0.060*	
C12	0.0223 (2)	0.69854 (14)	0.5464 (3)	0.0432 (5)	
H12	−0.0425	0.7207	0.4968	0.052*	
C13	0.06184 (17)	0.61842 (13)	0.5017 (2)	0.0334 (4)	
H13	0.0245	0.5872	0.4198	0.040*	
C14	0.2736 (2)	0.54270 (14)	0.2906 (3)	0.0399 (5)	
C15	0.3426 (6)	0.6209 (6)	0.1016 (5)	0.0951 (18)	0.547 (7)
H15A	0.2725	0.6506	0.0820	0.114*	0.547 (7)
H15B	0.3437	0.5700	0.0320	0.114*	0.547 (7)
C16	0.4336 (5)	0.6826 (5)	0.0708 (8)	0.0951 (18)	0.547 (7)
H16A	0.4368	0.6899	−0.0411	0.143*	0.547 (7)
H16B	0.5018	0.6586	0.1154	0.143*	0.547 (7)
H16C	0.4208	0.7390	0.1183	0.143*	0.547 (7)
C15'	0.3639 (8)	0.6479 (5)	0.1291 (8)	0.0951 (18)	0.45
H15C	0.3928	0.7055	0.1613	0.114*	0.453 (7)
H15D	0.2916	0.6563	0.0758	0.114*	0.453 (7)
C16'	0.4377 (7)	0.6047 (6)	0.0203 (7)	0.0951 (18)	0.45
H16D	0.4374	0.6381	−0.0760	0.143*	0.453 (7)
H16E	0.4124	0.5456	−0.0029	0.143*	0.453 (7)
H16F	0.5110	0.6025	0.0698	0.143*	0.453 (7)
C17	0.4187 (2)	0.39617 (19)	0.2990 (3)	0.0540 (6)	
H17A	0.4443	0.3364	0.2905	0.081*	
H17B	0.4780	0.4332	0.3402	0.081*	
H17C	0.3919	0.4172	0.1965	0.081*	

### Atomic displacement parameters (Å^2^)

**Table:**

	*U*^11^	*U*^22^	*U*^33^	*U*^12^	*U*^13^	*U*^23^
K1	0.0625 (4)	0.0453 (3)	0.0344 (3)	0.0047 (2)	0.0035 (2)	0.0039 (2)
N1	0.0531 (12)	0.0367 (9)	0.0414 (11)	0.0073 (8)	0.0011 (9)	0.0112 (8)
O2	0.0589 (10)	0.0395 (8)	0.0335 (8)	0.0062 (7)	0.0046 (7)	0.0133 (6)
C3	0.0453 (12)	0.0308 (9)	0.0259 (9)	−0.0015 (8)	−0.0034 (8)	0.0037 (7)
O3	0.0528 (10)	0.0494 (9)	0.0308 (8)	0.0095 (7)	0.0106 (7)	0.0049 (7)
C3A	0.0375 (10)	0.0257 (9)	0.0285 (9)	0.0010 (7)	−0.0010 (8)	0.0029 (7)
C4	0.0349 (10)	0.0243 (8)	0.0233 (8)	−0.0009 (7)	−0.0003 (7)	0.0012 (7)
C5	0.0368 (10)	0.0295 (9)	0.0307 (10)	−0.0032 (8)	0.0019 (8)	0.0003 (7)
O4	0.0706 (12)	0.0517 (10)	0.0373 (9)	−0.0031 (9)	−0.0058 (8)	0.0109 (7)
O5	0.0561 (12)	0.0650 (12)	0.0866 (16)	−0.0045 (9)	0.0216 (11)	0.0378 (11)
C6	0.0355 (11)	0.0332 (10)	0.0348 (11)	0.0016 (8)	0.0040 (8)	−0.0018 (8)
O6	0.0453 (9)	0.0297 (7)	0.0405 (8)	0.0019 (6)	−0.0035 (7)	−0.0067 (6)
C7	0.0389 (12)	0.0365 (11)	0.0437 (12)	0.0074 (9)	0.0003 (9)	0.0036 (9)
C7A	0.0398 (11)	0.0280 (9)	0.0341 (10)	0.0006 (8)	−0.0045 (8)	0.0030 (8)
C8	0.0397 (11)	0.0247 (9)	0.0242 (9)	−0.0012 (7)	0.0020 (8)	0.0009 (7)
C9	0.0575 (14)	0.0371 (11)	0.0397 (12)	0.0037 (10)	−0.0121 (10)	−0.0069 (9)
C10	0.0815 (19)	0.0385 (12)	0.0438 (13)	−0.0020 (12)	−0.0045 (13)	−0.0142 (10)
C11	0.0770 (18)	0.0297 (10)	0.0443 (13)	0.0099 (11)	0.0132 (12)	−0.0003 (9)
C12	0.0544 (14)	0.0341 (11)	0.0418 (12)	0.0109 (9)	0.0086 (10)	0.0105 (9)
C13	0.0419 (11)	0.0301 (9)	0.0284 (10)	0.0015 (8)	0.0033 (8)	0.0058 (8)
C14	0.0526 (13)	0.0326 (10)	0.0365 (11)	0.0013 (9)	0.0160 (10)	0.0047 (8)
C15	0.074 (2)	0.115 (4)	0.100 (3)	−0.008 (2)	0.026 (2)	0.065 (3)
C16	0.074 (2)	0.115 (4)	0.100 (3)	−0.008 (2)	0.026 (2)	0.065 (3)
C15'	0.074 (2)	0.115 (4)	0.100 (3)	−0.008 (2)	0.026 (2)	0.065 (3)
C16'	0.074 (2)	0.115 (4)	0.100 (3)	−0.008 (2)	0.026 (2)	0.065 (3)
C17	0.0547 (15)	0.0561 (15)	0.0537 (16)	0.0096 (12)	0.0215 (12)	0.0014 (12)

### Geometric parameters (Å, °)

**Table:**

K1—O3^i^	2.6059 (17)	O6—H6O	0.9092
K1—O4	2.688 (2)	C7—C7A	1.483 (3)
K1—O3^ii^	2.7010 (16)	C7—H7A	0.9900
K1—O6	2.8042 (16)	C7—H7B	0.9900
K1—O2^ii^	3.0285 (18)	C8—C13	1.380 (3)
K1—C3^ii^	3.299 (2)	C8—C9	1.384 (3)
K1—C12^i^	3.372 (2)	C9—C10	1.390 (3)
K1—C11^i^	3.411 (3)	C9—H9	0.9500
K1—K1^iii^	3.9778 (11)	C10—C11	1.369 (4)
N1—C7A	1.318 (3)	C10—H10	0.9500
N1—O2	1.430 (3)	C11—C12	1.368 (4)
O2—C3	1.387 (2)	C11—K1^i^	3.411 (3)
O2—K1^iv^	3.0285 (18)	C11—H11	0.9500
C3—O3	1.245 (3)	C12—C13	1.395 (3)
C3—C3A	1.380 (3)	C12—K1^i^	3.372 (2)
C3—K1^iv^	3.299 (2)	C12—H12	0.9500
O3—K1^i^	2.6059 (17)	C13—H13	0.9500
O3—K1^iv^	2.7011 (16)	C15—C16	1.520 (3)
C3A—C7A	1.395 (3)	C15—H15A	0.9900
C3A—C4	1.499 (3)	C15—H15B	0.9900
C4—C8	1.521 (3)	C16—H16A	0.9800
C4—C5	1.541 (3)	C16—H16B	0.9800
C4—H4	1.0000	C16—H16C	0.9800
C5—C14	1.513 (3)	C15'—C16'	1.522 (3)
C5—C6	1.560 (3)	C15'—H15C	0.9900
C5—H5	1.0000	C15'—H15D	0.9900
O4—C14	1.187 (3)	C16'—H16D	0.9800
O5—C14	1.332 (3)	C16'—H16E	0.9800
O5—C15'	1.446 (3)	C16'—H16F	0.9800
O5—C15	1.462 (3)	C17—H17A	0.9800
C6—O6	1.422 (3)	C17—H17B	0.9800
C6—C17	1.523 (3)	C17—H17C	0.9800
C6—C7	1.532 (3)		
			
O3^i^—K1—O4	77.88 (6)	O6—C6—C7	109.96 (17)
O3^i^—K1—O3^ii^	82.92 (5)	C17—C6—C7	110.03 (19)
O4—K1—O3^ii^	82.45 (6)	O6—C6—C5	106.27 (16)
O3^i^—K1—O6	131.05 (5)	C17—C6—C5	112.27 (18)
O4—K1—O6	67.46 (5)	C7—C6—C5	109.00 (17)
O3^ii^—K1—O6	123.35 (5)	C6—O6—K1	135.57 (12)
O3^i^—K1—O2^ii^	128.12 (5)	C6—O6—H6O	110.0
O4—K1—O2^ii^	87.56 (6)	K1—O6—H6O	110.1
O3^ii^—K1—O2^ii^	45.63 (5)	C7A—C7—C6	111.18 (17)
O6—K1—O2^ii^	84.83 (5)	C7A—C7—H7A	109.4
O3^i^—K1—C3^ii^	103.30 (5)	C6—C7—H7A	109.4
O4—K1—C3^ii^	81.74 (6)	C7A—C7—H7B	109.4
O3^ii^—K1—C3^ii^	21.07 (5)	C6—C7—H7B	109.4
O6—K1—C3^ii^	104.65 (5)	H7A—C7—H7B	108.0
O2^ii^—K1—C3^ii^	24.85 (5)	N1—C7A—C3A	113.3 (2)
O3^i^—K1—C12^i^	96.90 (5)	N1—C7A—C7	123.19 (19)
O4—K1—C12^i^	114.94 (6)	C3A—C7A—C7	123.52 (19)
O3^ii^—K1—C12^i^	162.27 (6)	C13—C8—C9	118.26 (19)
O6—K1—C12^i^	69.74 (6)	C13—C8—C4	119.40 (17)
O2^ii^—K1—C12^i^	133.79 (5)	C9—C8—C4	122.23 (18)
C3^ii^—K1—C12^i^	156.31 (6)	C8—C9—C10	120.5 (2)
O3^i^—K1—C11^i^	100.67 (6)	C8—C9—H9	119.7
O4—K1—C11^i^	138.20 (6)	C10—C9—H9	119.7
O3^ii^—K1—C11^i^	139.26 (6)	C11—C10—C9	120.6 (2)
O6—K1—C11^i^	84.75 (6)	C11—C10—H10	119.7
O2^ii^—K1—C11^i^	121.58 (5)	C9—C10—H10	119.7
C3^ii^—K1—C11^i^	137.22 (6)	C12—C11—C10	119.6 (2)
C12^i^—K1—C11^i^	23.26 (6)	C12—C11—K1^i^	76.75 (14)
O3^i^—K1—K1^iii^	42.37 (3)	C10—C11—K1^i^	86.48 (16)
O4—K1—K1^iii^	76.89 (4)	C12—C11—H11	120.2
O3^ii^—K1—K1^iii^	40.55 (4)	C10—C11—H11	120.2
O6—K1—K1^iii^	143.46 (4)	K1^i^—C11—H11	106.8
O2^ii^—K1—K1^iii^	85.96 (3)	C11—C12—C13	120.0 (2)
C3^ii^—K1—K1^iii^	61.12 (4)	C11—C12—K1^i^	79.98 (14)
C12^i^—K1—K1^iii^	136.51 (5)	C13—C12—K1^i^	86.09 (12)
C11^i^—K1—K1^iii^	129.48 (5)	C11—C12—H12	120.0
C7A—N1—O2	104.50 (17)	C13—C12—H12	120.0
C3—O2—N1	109.00 (16)	K1^i^—C12—H12	104.0
C3—O2—K1^iv^	88.57 (12)	C8—C13—C12	121.0 (2)
N1—O2—K1^iv^	160.52 (12)	C8—C13—H13	119.5
O3—C3—C3A	135.81 (19)	C12—C13—H13	119.5
O3—C3—O2	116.73 (19)	O4—C14—O5	122.5 (2)
C3A—C3—O2	107.45 (18)	O4—C14—C5	125.5 (2)
O3—C3—K1^iv^	51.27 (11)	O5—C14—C5	112.0 (2)
C3A—C3—K1^iv^	168.76 (15)	O5—C15—C16	108.3 (3)
O2—C3—K1^iv^	66.58 (11)	O5—C15—H15A	110.0
C3—O3—K1^i^	151.29 (14)	C16—C15—H15A	110.0
C3—O3—K1^iv^	107.66 (13)	O5—C15—H15B	110.0
K1^i^—O3—K1^iv^	97.08 (5)	C16—C15—H15B	110.0
C3—C3A—C7A	105.72 (18)	H15A—C15—H15B	108.4
C3—C3A—C4	130.19 (18)	O5—C15'—C16'	108.5 (3)
C7A—C3A—C4	123.71 (18)	O5—C15'—H15C	110.0
C3A—C4—C8	112.89 (15)	C16'—C15'—H15C	110.0
C3A—C4—C5	108.52 (16)	O5—C15'—H15D	110.0
C8—C4—C5	113.49 (16)	C16'—C15'—H15D	110.0
C3A—C4—H4	107.2	H15C—C15'—H15D	108.4
C8—C4—H4	107.2	C15'—C16'—H16D	109.5
C5—C4—H4	107.2	C15'—C16'—H16E	109.5
C14—C5—C4	110.71 (17)	H16D—C16'—H16E	109.5
C14—C5—C6	109.48 (17)	C15'—C16'—H16F	109.5
C4—C5—C6	110.82 (16)	H16D—C16'—H16F	109.5
C14—C5—H5	108.6	H16E—C16'—H16F	109.5
C4—C5—H5	108.6	C6—C17—H17A	109.5
C6—C5—H5	108.6	C6—C17—H17B	109.5
C14—O4—K1	131.21 (15)	H17A—C17—H17B	109.5
C14—O5—C15'	125.5 (5)	C6—C17—H17C	109.5
C14—O5—C15	107.7 (3)	H17A—C17—H17C	109.5
C15'—O5—C15	21.4 (5)	H17B—C17—H17C	109.5
O6—C6—C17	109.24 (19)		
			
C7A—N1—O2—C3	1.0 (2)	O2^ii^—K1—O6—C6	92.04 (18)
C7A—N1—O2—K1^iv^	−152.5 (3)	C3^ii^—K1—O6—C6	76.92 (18)
N1—O2—C3—O3	177.58 (19)	C12^i^—K1—O6—C6	−127.24 (18)
K1^iv^—O2—C3—O3	−10.98 (19)	C11^i^—K1—O6—C6	−145.53 (18)
N1—O2—C3—C3A	−1.6 (2)	K1^iii^—K1—O6—C6	16.0 (2)
K1^iv^—O2—C3—C3A	169.83 (15)	O6—C6—C7—C7A	−68.8 (2)
N1—O2—C3—K1^iv^	−171.44 (16)	C17—C6—C7—C7A	170.8 (2)
C3A—C3—O3—K1^i^	−19.9 (5)	C5—C6—C7—C7A	47.3 (2)
O2—C3—O3—K1^i^	161.3 (2)	O2—N1—C7A—C3A	0.0 (3)
K1^iv^—C3—O3—K1^i^	148.3 (4)	O2—N1—C7A—C7	179.66 (19)
C3A—C3—O3—K1^iv^	−168.2 (2)	C3—C3A—C7A—N1	−0.9 (3)
O2—C3—O3—K1^iv^	12.9 (2)	C4—C3A—C7A—N1	−174.48 (19)
O3—C3—C3A—C7A	−177.4 (3)	C3—C3A—C7A—C7	179.4 (2)
O2—C3—C3A—C7A	1.5 (2)	C4—C3A—C7A—C7	5.8 (3)
K1^iv^—C3—C3A—C7A	57.8 (8)	C6—C7—C7A—N1	161.3 (2)
O3—C3—C3A—C4	−4.5 (4)	C6—C7—C7A—C3A	−19.0 (3)
O2—C3—C3A—C4	174.50 (19)	C3A—C4—C8—C13	−126.32 (19)
K1^iv^—C3—C3A—C4	−129.3 (7)	C5—C4—C8—C13	109.7 (2)
C3—C3A—C4—C8	40.0 (3)	C3A—C4—C8—C9	49.8 (3)
C7A—C3A—C4—C8	−148.17 (19)	C5—C4—C8—C9	−74.2 (3)
C3—C3A—C4—C5	166.7 (2)	C13—C8—C9—C10	−0.5 (4)
C7A—C3A—C4—C5	−21.5 (3)	C4—C8—C9—C10	−176.6 (2)
C3A—C4—C5—C14	172.36 (16)	C8—C9—C10—C11	0.7 (4)
C8—C4—C5—C14	−61.3 (2)	C9—C10—C11—C12	0.3 (4)
C3A—C4—C5—C6	50.7 (2)	C9—C10—C11—K1^i^	72.9 (3)
C8—C4—C5—C6	177.04 (16)	C10—C11—C12—C13	−1.4 (4)
O3^i^—K1—O4—C14	137.7 (2)	K1^i^—C11—C12—C13	−79.5 (2)
O3^ii^—K1—O4—C14	−138.0 (2)	C10—C11—C12—K1^i^	78.1 (2)
O6—K1—O4—C14	−7.0 (2)	C9—C8—C13—C12	−0.7 (3)
O2^ii^—K1—O4—C14	−92.4 (2)	C4—C8—C13—C12	175.58 (18)
C3^ii^—K1—O4—C14	−116.7 (2)	C11—C12—C13—C8	1.7 (3)
C12^i^—K1—O4—C14	45.6 (2)	K1^i^—C12—C13—C8	−74.42 (19)
C11^i^—K1—O4—C14	45.2 (3)	K1—O4—C14—O5	148.93 (19)
K1^iii^—K1—O4—C14	−178.8 (2)	K1—O4—C14—C5	−30.2 (3)
C14—C5—C6—O6	−70.3 (2)	C15'—O5—C14—O4	0.8 (5)
C4—C5—C6—O6	52.1 (2)	C15—O5—C14—O4	−12.5 (5)
C14—C5—C6—C17	49.0 (3)	C15'—O5—C14—C5	−180.0 (4)
C4—C5—C6—C17	171.44 (19)	C15—O5—C14—C5	166.8 (5)
C14—C5—C6—C7	171.22 (18)	C4—C5—C14—O4	−42.8 (3)
C4—C5—C6—C7	−66.4 (2)	C6—C5—C14—O4	79.6 (3)
C17—C6—O6—K1	−89.3 (2)	C4—C5—C14—O5	137.97 (19)
C7—C6—O6—K1	149.81 (14)	C6—C5—C14—O5	−99.6 (2)
C5—C6—O6—K1	32.0 (2)	C14—O5—C15—C16	176.7 (6)
O3^i^—K1—O6—C6	−45.9 (2)	C15'—O5—C15—C16	27.6 (11)
O4—K1—O6—C6	2.54 (17)	C14—O5—C15'—C16'	−104.3 (7)
O3^ii^—K1—O6—C6	66.21 (19)	C15—O5—C15'—C16'	−67.4 (14)

Symmetry codes: (i) −*x*, −*y*+1, −*z*+1; (ii) *x*, *y*, *z*−1; (iii) −*x*, −*y*+1, −*z*; (iv) *x*, *y*, *z*+1.

### Hydrogen-bond geometry (Å, °)

**Table:**

*D*—H···*A*	*D*—H	H···*A*	*D*···*A*	*D*—H···*A*
O6—H6O···N1^v^	0.91	1.88	2.784 (3)	177
O6—H6O···O2^v^	0.91	2.55	3.336 (3)	145

Symmetry codes: (v) *x*, −*y*+1/2, *z*−1/2.
